# Low Expression of miR-466f-3p Sustains Epithelial to Mesenchymal Transition in Sonic Hedgehog Medulloblastoma Stem Cells Through Vegfa-Nrp2 Signaling Pathway

**DOI:** 10.3389/fphar.2018.01281

**Published:** 2018-11-12

**Authors:** Zein Mersini Besharat, Claudia Sabato, Agnese Po, Francesca Gianno, Luana Abballe, Maddalena Napolitano, Evelina Miele, Felice Giangaspero, Alessandra Vacca, Giuseppina Catanzaro, Elisabetta Ferretti

**Affiliations:** ^1^Department of Experimental Medicine, Sapienza University, Rome, Italy; ^2^Department of Molecular Medicine, Sapienza University, Rome, Italy; ^3^Center for Life NanoScience@Sapienza, Istituto Italiano di Tecnologia, Rome, Italy; ^4^Department of Radiological, Oncological and Pathological Science, Sapienza University, Rome, Italy; ^5^Department of Hematology/Oncology and Stem Cell Transplantation, Bambino Gesù Children’s Hospital, Istituto di Ricovero e Cura a Carattere Scientifico, Rome, Italy; ^6^IRCCS Neuromed, Isernia, Italy

**Keywords:** medulloblastoma, sonic hedgehog medulloblastoma cancer stem cells, epithelial to mesenchymal transition, vegfa, Nrp2, miR-466f-3p

## Abstract

High-throughput analysis has improved the knowledge of medulloblastoma (MB), the leading cause of cancer related death in children, allowing a better comprehension of the key molecular pathways in MB pathogenesis. However, despite these advances, 30% of patients still die from the disease and survivors face severe long-term side effects. Cancer stem cells (CSCs) represent a subset of cells that not only drive tumorigenesis, but are also one of the main determinants of chemoresistance. Epithelial mesenchymal transition (EMT) is a hallmark of cancer and up to now few data is available in MB. To give insight into the role of the EMT process in maintaining the mesenchymal phenotype of CSCs, we analyzed the expression of EMT related transcripts and microRNAs in these cells. We firstly isolated CSCs from Sonic Hedgehog (SHH) MB derived from Ptch1 heterozygous mice and compared their expression level of EMT-related transcripts and microRNAs with cerebellar NSCs. We identified two molecules linked to SHH and EMT, Vegfa and its receptor Nrp2, over-expressed in SHH MB CSCs. Inhibition of Vegfa showed impairment of cell proliferation and self-renewal ability of CSCs concurrent with an increase of the expression of the EMT gene, E-cadherin, and a decrease of the EMT marker, Vimentin. Moreover, among deregulated microRNAs, we identified miR-466f-3p, a validated inhibitor of both Vegfa and Nrp2. These results allowed us to describe a new EMT molecular network, involving the down-regulation of miR-466f-3p together with the concordant up-regulation of Vegfa and Nrp2, that sustains the mesenchymal phenotype of SHH MB CSCs.

## Introduction

Medulloblastoma (MB) is the most common malignant brain tumor of the pediatric age and a leading cause of cancer related morbidity and mortality (Northcott et al., 2012). Despite the fact that multimodal aggressive therapy has improved MB outcome, 30% of patients still die of disease and about 40% face tumor recurrence. Moreover, survivors frequently develop long-term severe side effects (Wang et al., 2018). In recent years, high-throughput studies have been conducted to better understand MB biology and key signaling pathways that could be addressed to reach a better management of MB patients. These studies allowed the recent WHO 2016 subgrouping of MB (Louis et al., 2016), identifying five subgroups: WNT activated, SHH activated P53 wild-type, SHH activated P53 mutant, non-WNT/non-SHH Group 3, non-WNT/non-SHH Group 4 (Louis et al., 2016) and more recently 12 molecular subtypes (Cavalli et al., 2017; Northcott et al., 2017). In this context, SHH subgroups account for about 30% of cases (Cavalli et al., 2017) and they are the most common MB subtypes in infants and adults (Kool et al., 2012).

Cancer stem cells (CSCs) have been described in MB (Lee et al., 2005; Po et al., 2010; Manoranjan et al., 2013; Mastronuzzi et al., 2014). CSCs may arise from the malignant transformation of neural stem cells (NSCs) and represent a reservoir for cancer maintenance and progression (Northcott et al., 2012). We isolated CSCs from Ptch heterozygous mice, a model of the SHH MB subgroup (Goodrich et al., 1997; Po et al., 2010; Wu et al., 2011; Ronci et al., 2015), and performed transcriptome analysis of both SHH MB CSCs and NSCs isolated from postnatal murine cerebellum for comparison. Among the identified transcripts that characterize SHH MB CSCs, genes involved in the epithelial-mesenchymal transition (EMT) were highly represented and some of them resulted significantly differentially expressed between SHH MB CSCs and NSCs. EMT is characterized by the loss of epithelial characteristics and the acquisition of mesenchymal properties and previous studies linked a shift toward mesenchymal properties to metastatic progression and acquisition of stemness features (Taube et al., 2010). Among deregulated markers of EMT in SHH MB CSCs, we focused on Vegfa and Nrp2 that have been described as pivotal players in tumorigenesis and in maintaining stemness and proliferation (Prud’homme and Glinka, 2012; Fantozzi et al., 2014). We show that both Vegfa and Nrp2 correlate with stemness features in SHH MB CSCs and that Vegfa inhibition determines an increase in the expression of the epithelial marker E-cadherin, a reduction of the mesenchymal marker Vimentin and an impairment of self-renewal. Moreover, since accumulating evidence indicate a crucial role of microRNAs in the regulation of a variety of biological processes, including EMT (Markopoulos et al., 2017), we also focused our attention on the microRNAs differentially expressed between NSCs and SHH MB CSCs and involved in EMT identifying an epigenetic circuitry that sustains the mesenchymal phenotype of SHH MB CSCs.

## Materials and Methods

Unless otherwise indicated, media and supplements were purchased from Gibco/Invitrogen (Carlsbad, CA) and chemicals from Sigma-Aldrich (St. Louis, MO). Animal experiments were approved by local ethic authorities and conducted in accordance with Italian Governing Law (D.lgs 26/2014; Prot. no. 03/2013).

### Cell Culture, Treatments, Proliferation and Oncosphere-Forming Assays

SHH MB CSCs were derived from spontaneous tumours arisen in Ptch1 + /− mice and maintained as previously described (Po et al., 2010). To induce differentiation, cells were plated on D-poly-lysine coated supports and treated for 48 h with 2 μM retinoic acid (Ronci et al., 2015). BrdU incorporation was used to evaluate SHH MB CSCs proliferation before (CSC) and after differentiation (CSC-diff) (Miele et al., 2017a). Pharmacological inhibition of Vegfa was induced by treating SHH MB CSC cells for 72 h with 10, 20, 40 and 60 ng/ml anti-Vegf (MAB 293, R&D Systems). Synthetic miR-466f-3p (4464066, Thermo Fisher Scientific) or negative control (miRIDIAN CN-001000-01; Dharmacon) were used as previously described (Catanzaro et al., 2018). Cell proliferation was evaluated by trypan blue exclusion assay (Catanzaro et al., 2018). Oncosphere-forming assay of SHH MB CSC was performed as previously described (Po et al., 2010). Unpaired *t*-test of three independent experiments was performed using GraphPad Prism Software version 6.0 (CA, United States), *p*–values < 0.05 were considered statistically significant.

### RNA Extraction, miRNA and mRNA Sequencing

Three biological replicates of SHH MB CSCs were subjected to miRNA-sequencing or mRNA sequencing, quality control, mapping, quantification and differential expression analysis was performed between SHH MB CSCs and NSCs (Besharat et al., 2018; Po et al., 2018).

### Immunofluorescence

Immunofluorescence studies were performed according to standard procedures (Catanzaro et al., 2011) using the following primary antibodies: anti-Vegfa Clone VG1 (05-1117, Millipore), anti-Nrp2 H-300 (sc-5542), anti-Nanog (8600S, Cell Signaling), anti-NeuN (MAB377, Millipore).

### Immunochemical Analysis

Western blotting was performed as previously described (Miele et al., 2017b) using the following primary antibodies: anti-E-cadherin (610181, BD Biosciences), anti-Vimentin (92547, Abcam), anti-Vegfa Clone VG1 (05-1117, Millipore), anti-Nrp2 (ab 185710, Abcam), anti-β-actin I-19 (sc-1616, Santa Cruz).

## Results

### EMT Related Transcripts Characterize SHH MB CSC

**FIGURE 1:**
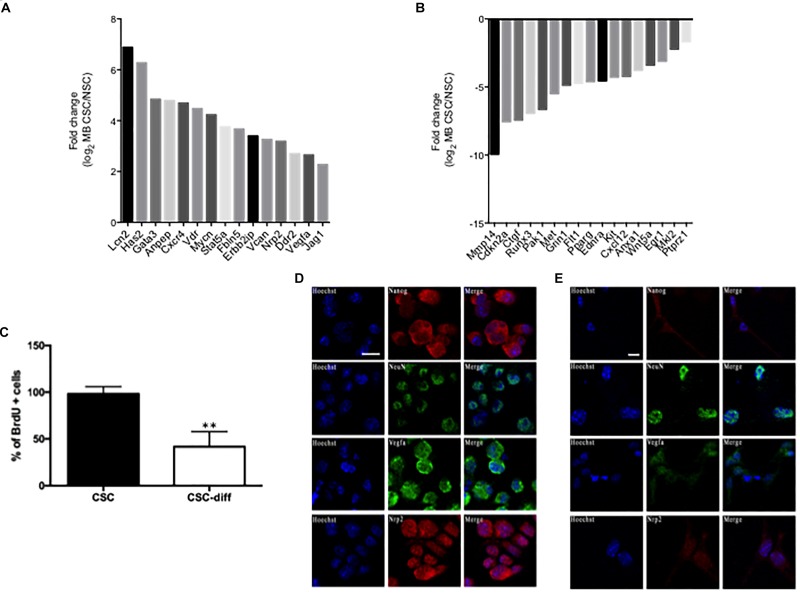
EMT-related characterization of SHH MB CSC. **(A,B)** Statistically significant up-regulated **(A)** and down-regulated **(B)** EMT-related genes in SHH MB CSC vs. NSC. **(C)** Bromodeoxyuridine (BrdU) uptake in SHH MB CSC before (CSC) and after differentiation (CSC-diff). ^∗∗^ indicates *p* < 0.01 vs. CSC-diff. **(D,E)** Immunofluorescence staining of the stemness marker Nanog and the neuronal marker NeuN, and of the EMT-related markers, Vegfa and Nrp2, in SHH MB CSC before **(D)** and after 48 h differentiation with 2 μM RA **(E)**. SHH MB CSCs express higher levels of Vegfa and Nrp2 than SHH MB CSC-diff. The differentiated status of SHH MB CSC was confirmed by the down-regulation of Nanog and the up-regulation of NeuN in SHH MB CSC after 48 h of differentiation. Bars, 10 μm.

We recently conducted small RNA and trascriptome sequencing on SHH MB CSCs and NSCs (Besharat et al., 2018; Po et al., 2018). In this study, we focused on the EMT related RNAs that characterize SHH MB CSCs compared to NSCs, querying the differentially expressed transcripts listed in the EMT gene database dbEMT (Zhao et al., 2015). Deregulated transcripts are shown in Figure 1, specifically, 17 mRNA resulted down-regulated and 15 up-regulated in SHH MB CSCs when compared with NSCs (Figures 1A,B). Since we were interested in the mechanisms that could regulate EMT in SHH MB CSCs, we used qPCR to validate the up-regulated transcripts (data not shown). Among them, we focused on Nrp2 and Vegfa, whose role in maintaining stemness and proliferation in lung cancer stem cells we recently demonstrated (Po et al., 2017). To investigate whether they correlate with stemness features in the context of SHH MB, the protein expression of Vegfa and Nrp2 in SHH MB CSCs was evaluated before (CSC) and after differentiation (CSC-diff). Differentiated cells are characterized by a lower proliferative rate, as shown by the reduction in BrdU incorporation (Figure 1C), and less aggressive behavior in comparison with SHH MB CSCs (Morelli et al., 2012). We observed a reduction in the protein level of Vegfa and Nrp2 in SHH MB CSC after 48h of Retinoic Acid (RA)-induced differentiation (Figures 1D,E). The differentiated status of SHH MB CSCs was confirmed by the decrease of the stemness marker Nanog and the increase of the neuronal marker NeuN (Preusser et al., 2006) after differentiation (Figure 1E).

### EMT-Related Circuitry Activation in SHH MB CSCs

**FIGURE 2:**
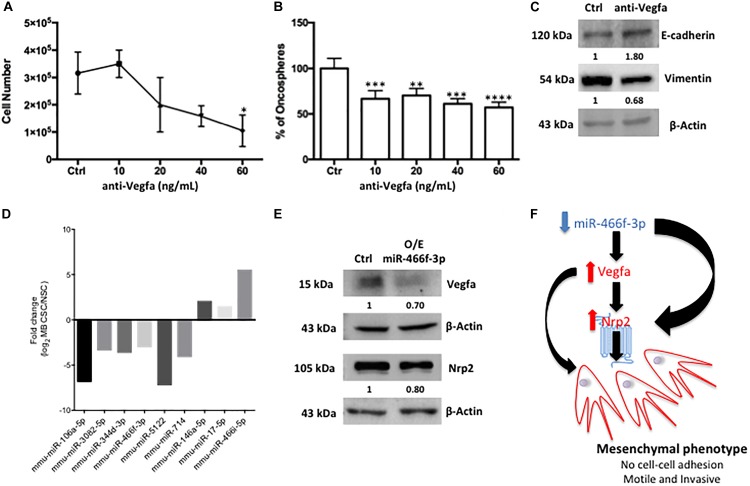
Pharmacologic inhibition of Vegfa signaling and EMT-related microRNA/mRNA network in SHH MB CSC. **(A–C)** After 72 h of Vegfa inhibition cell proliferation **(A)** and self-renewal **(B)** were impaired. Western blot analysis was performed with the more effective anti-Vegfa concentration (60 ng/mL) and showed an increase in the expression of the epithelial marker E-cadherin and a reduction in the level of the mesenchymal marker Vimentin in respect to untreated SHH MB CSC (Ctrl) **(C)**. ^∗^ indicates *p* < 0.05, ^∗∗^*p* < 0.01, ^∗∗∗^*p* < 0.001, and ^∗∗∗∗^*p* < 0.0001 vs. Ctrl **(D)** Statistically significant deregulated EMT-related microRNAs in SHH MB CSC vs. NSC that target the previously identified deregulated transcripts. **(E)** Western blot analysis was performed after over-expression of 20 nM of miR-466f-3p for 48 h and demonstrated a reduction of Vegfa and Nrp2 expression in respect to untreated SHH MB CSCs (Ctrl) **(F)** The miR-466f-3p/Vegfa/Nrp2 circuitry sustaining the mesenchymal phenotype of SHH MB CSC.

To unravel the role of Vegfa in SHH MB CSCs, we performed pharmacological modulation by using a blocking antibody. We focused on Vegfa since it acts as a ligand for Nrp2, therefore its inhibition likely determines an inhibition of the Nrp2-mediated signaling. After 72h, Vegfa inhibition induced an impairment of both cell proliferation (Figure 2A) and clonogenic ability (Figure 2B) of SHH MB CSCs. Concordantly, we observed an increase in the epithelial marker E-cadherin and a reduction in the mesenchymal marker Vimentin (Figure 2C), indicating that Vegfa is involved in the induction of EMT in SHH MB CSCs. Subsequently, we investigated an EMT-related network involving both mRNAs and microRNAs that could characterize SHH MB CSCs. With this aim, we compared the miRnome of SHH MB CSC and NSC (Besharat et al., 2018) focusing on the differentially expressed microRNAs that target the previously identified deregulated transcripts (Figures 1A,B) as reported in miRTarBase (Chou et al., 2017). Specifically, we observed six down-regulated and three up-regulated microRNAs in SHH MB CSCs (Figure 2D), that we validated by using qPCR (data not shown). Among them, miR-106a targeted only Vegfa, while miR-3082 and miR-5122 targeted only Nrp2. Interestingly, miR-466f-3p targeted both Vegfa and Nrp2. To confirm the regulation of Vegfa and Nrp2 by miR-466f-3p in SHH MB CSCs, we overexpressed this microRNA obtaining a reduction of Vegfa and Nrp2 levels (Figure 2E). These results indicate the existence of a functional circuitry between these molecules (Figure 2F) involved in the induction of EMT in SHH MB CSCs.

## Discussion

MB is the most frequent malignant childhood brain tumor and CSCs have been an important focus for researchers. On the basis of the cancer stem cell hypothesis, CSCs represent a subset of cells within the tumor with the ability to proliferate and maintain tumor growth (Manoranjan et al., 2013). CSCs have been identified in all MB subtypes and are responsible for therapeutic resistance and invasion (Kumar et al., 2017). The cellular origin and stage of differentiation are of pivotal importance in determining the tumor phenotype in MB (Lin et al., 2016). Specifically, in SHH MB the deregulated SHH signaling works as a potent mitogen to induce the proliferation of the granule neuron precursors, where a specific deletion of Ptch1 or Smo activation determines MB in mouse models (Schüller et al., 2008). Some reports described the importance of EMT in increasing the migratory and invasive abilities of MB cells (Asuthkar et al., 2011; Gupta et al., 2011; Singh et al., 2016; Ferrucci et al., 2018; Gao et al., 2018), however, no report has addressed the EMT phenomenon in MB CSCs. Since CSCs represent interesting candidates to determine MB migration and invasion, we examined the role of EMT in CSCs belonging to a SHH MB model, derived from specific transgenic mice haploinsufficient for Ptch1 (Ptch1 + /−).

Firstly, we evaluated the expression of EMT-related genes in SHH MB CSC in comparison with NSC cells derived from postnatal cerebellum, where medulloblastoma arises.

Among the 15 up-regulated EMT-related transcripts we focused on Vegfa, which is one of the main mammalian HH target genes (Kumar et al., 2017), and Nrp2, a transmembrane protein required for HH signal transduction (Gephart et al., 2013). Vegfa has been described in solid cancers as a crucial determinant of the increase in tumorigenicity of cells that undergo EMT, both inducing angiogenetic (Fantozzi et al., 2014) and non-angiogenetic events (Gonzalez-Moreno et al., 2010). Moreover, Vegfa has been demonstrated to increase the tumor-initiating stem cell population, to induce EMT and metastasis (Kim et al., 2017), suggesting a strong link between CSC and EMT. On this basis, we inhibited Vegfa and evaluated the proliferative and self-renewal ability of CSCs and the modulation of two critical markers of the EMT process. Vegfa inhibition induced a reduction in cell growth and clonogenic ability of CSCs and an up-regulation of the cell adhesion molecule E-cadherin, paralleled by a down-regulation of the mesenchymal marker, Vimentin. These results indicate that Vegfa is involved both in maintaining the stem cells niche and in promoting cancer invasion and metastasis by controlling the EMT program. Also Nrp2 is involved in EMT and has been reported as up-regulated both in hepatocellular carcinoma and in lung cancer cells after EMT induction by TGF-β1 (Nasarre et al., 2013; Wittmann et al., 2015). Nrp2 inhibition in a cellular mouse model of SHH MB decreased tumor growth both *in vitro* and *in vivo* and the consequent mortality (Gephart et al., 2013). Since the importance of microRNAs has been well documented in cancers and we were interested in the identification of possible networks connecting Vegfa and/or Nrp2 with the EMT phenomenon in SHH MB CSC, we extended the analysis to the EMT related microRNAs deregulated between SHH MB CSC and NSC. Interestingly we identified a microRNA, miR-466f-3p, that targeted both Vegfa and Nrp2. Knowledge about this microRNA is scarce and scant. In 2011 Zheng demonstrated that the miR-466 group is contained in the intron of *Sfmbt2* (Zheng et al., 2011), while in 2012 Hunsberger et al. showed an increase in miR-466f expression in a rat model of middle cerebral artery occlusion (Hunsberger et al., 2012). The low level of miR-466f-3p in SHH MB CSCs is involved in the more mesenchymal phenotype of these cells in respect to NSCs. In fact the down-regulation of miR-466f-3p is related with an increase of the Nrp2 level or of its ligand, Vegfa, with a consequent increase in Nrp2 activation. In both cases the final result sustains the mesenchymal phenotype of SHH MB CSCs. In summary, our study provides novel evidence of an epigenetic mechanism that sustains EMT related genes in SHH MB CSC, however, future investigations and additional studies are needed to better clarify the role of the miR-466f-3p/Vegfa/Nrp2 circuitry.

## Author Contributions

ZB, CS, FGianno, EM, and LA performed the experiments and analysis. FGiangaspero, AV, and MN contributed reagents and analytical tools. GC and AP conceived and designed the research. GC and EF wrote the paper. EF supervised the project. All authors contributed to the final version of the manuscript.

## Conflict of Interest Statement

The authors declare that the research was conducted in the absence of any commercial or financial relationships that could be construed as a potential conflict of interest. The handling editor declared a shared affiliation, though no other collaboration, with several of the authors EM, AP at the time of the review.
